# A first-line diagnostic assay for limb-girdle muscular dystrophy and other myopathies

**DOI:** 10.1186/s40246-016-0089-8

**Published:** 2016-09-27

**Authors:** Dorota Monies, Hindi N. Alhindi, Mohamed A. Almuhaizea, Mohamed Abouelhoda, Anas M. Alazami, Ewa Goljan, Banan Alyounes, Dyala Jaroudi, Abdulelah AlIssa, Khalid Alabdulrahman, Shazia Subhani, Mohamed El-Kalioby, Tariq Faquih, Salma M. Wakil, Nada A. Altassan, Brian F. Meyer, Saeed Bohlega

**Affiliations:** 1 Department of Genetics, Research Centre, King Faisal Specialist Hospital and Research Centre, PO Box 3354, Riyadh, 11211 Kingdom of Saudi Arabia; 2 Saudi Human Genome Program, King Abdulaziz City for Science and Technology, Riyadh, Saudi Arabia; 3 Department of Neuroscience, King Faisal Specialist Hospital and Research Centre, Riyadh, Saudi Arabia; 4 Department of Pathology and Laboratory Medicine, King Faisal Specialist Hospital and Research Centre, Riyadh, Saudi Arabia

**Keywords:** Neurological panel, Targeted resequencing, LGMD, Myopaties

## Abstract

**Background:**

Fifty random genetically unstudied families (limb-girdle muscular dystrophy (LGMD)/myopathy) were screened with a gene panel incorporating 759 OMIM genes associated with neurological disorders. Average coverage of the CDS and 10 bp flanking regions of genes was 99 %. All families were referred to the Neurosciences Clinic of King Faisal Specialist Hospital and Research Centre, Saudi Arabia. Patients presented with muscle weakness affecting the pelvic and shoulder girdle. Muscle biopsy in all cases showed dystrophic or myopathic changes. Our main objective was to evaluate a neurological gene panel as a first-line diagnostic test for LGMD/myopathies.

**Results:**

Our panel identified the mutation in 76 % of families (38/50; 11 novel). Thirty-four families had mutations in LGMD-related genes with four others having variants not typically associated with LGMD. The majority of cases had recessive inheritance with homoallelic pathogenic variants (97.4 %, 37/38), as expected considering the high rate of consanguinity in the study population. In one case, we detected a heterozygous mutation in *DNAJB* responsible for LGMD-1E. Our cohort included seven different subtypes of LGMD2. Mutations of *DYSF* were the most commonly identified cause of disease followed by that in *CAPN3* and *FKRP*. Non-LGMD myopathies were due to mutations in genes associated with congenital disorder of glycosylation (*ALG2*), rigid spine muscular dystrophy 1 (*SEPN1*), inclusion body myopathy2/Nonaka myopathy (*GNE*), and neuropathy (*WNK1*). Whole exome sequencing (WES) of patients who remained undiagnosed with the neurological panel did not improve our diagnostic yield.

**Conclusions:**

Our neurological panel achieved a high clinical sensitivity (76 %) and is an effective first-line laboratory test in patients with LGMD and other myopathies. This sensitive, cost-effective, and rapid assay significantly assists clinical practice especially in these phenotypically and genetically heterogeneous disorders. Moreover, the application of the American College of Medical Genetics (ACMG) and Association for Molecular Pathology (AMP) guidelines applied in the classification of variant pathogenecity provides a clear interpretation for physicians on the relevance of such findings.

**Electronic supplementary material:**

The online version of this article (doi:10.1186/s40246-016-0089-8) contains supplementary material, which is available to authorized users.

## Background

The original definition of limb-girdle muscular dystrophy (LGMD) as non-Duchenne with autosomal recessive inheritance was introduced in 1953 [1]. LGMD classification has rapidly evolved since that time. A major advance in neuromuscular disorders over the last three decades has been the identification of many genes underlying this group of heterogeneous diseases [2–4]. The LGMDs now vary widely in their genetics and also in clinical features ranging from very mild forms which allow patients to maintain a fairly normal life to much more severe deterioration of proximal limb muscles that causes dramatic physical weakness along with a shortened life-span [4, 5]. There are two major groups: LGMD1 and LGMD2 with autosomal dominant and autosomal recessive patterns of inheritance, respectively [6]. To date, there are at least 8 genes associated with LGMD1 (LGMD1A-1H) and 23 genes in which mutations lead to different subtypes of LGMD2 (LGMD2A-2W) [4] that continues to expand. This growing genetic heterogeneity highlights the problem of a very complex clinical diagnosis [7]. Current recommendations for diagnosis and management of LGMD are very complex and require access to multiple specialties including thorough clinical examination, laboratory testing, muscle imaging, histological appearances of muscle, and more [5]. Since there are overlapping phenotypes in LGMDs and a number of other myopathic disorders, a precise diagnosis without genetic testing is very difficult. As a consequence of this, many patients remain undiagnosed.

Next-generation sequencing (NGS) also known as massively parallel sequencing has ushered in a new era in molecular diagnostics. The availability of exome sequencing has been rapidly applied to clinical settings [8, 9]. Whiles the cost of a clinical-grade whole exome sequencing (WES) is still high and its interpretation very complex, an alternative approach to WES is the use of a NGS-based gene panel. This approach is particularly well suited for genetically and clinically heterogeneous conditions where the number and/or size of genes is too large and expensive to sequence one gene at a time [10]. In this study, we aimed to determine clinical sensitivity of a neurological gene panel for diagnosis of LGMD/myopathies on the basis that this would be a cheaper, more practical, and effective approach.

## Results

A total of 50 families were included in this study, of which 36 had an autosomal recessive pattern of inheritance with parental consanguinity. Mean age of disease onset for our patients was 10.6 years. Distribution of muscle weakness, age of onset, creatine kinase level, biopsy findings, and other clinical characteristics are presented in the supplementary data (see Additional file 1). Disease-causing mutations classified based on American College of Medical Genetics (ACMG)/Association for Molecular Pathology (AMP) guidelines were identified in 76 % of the study cohort (38/50 families). Novel mutations were present in 11 of these families (Table 1). The neurological panel revealed pathogenic variants in LGMD and non-LGMD myopathy related genes in 34 and 4 of our families, respectively (Table 2). The majority of our cases with pathogenic causal variants were homoallelic (97.4 %, 37/38), consistent with an autosomal recessive pattern of inheritance and as expected within a highly consanguineous population [11, 12]. We identified only one family with autosomal dominant inheritance, the result of a heterozygous mutation in *DNAJB* (LGMD type 1E)*,* present in two affected memebers (father and his son). The neurological panel detected seven subtypes of LGMD2, mutations of *DYSF* (LGMD2???) being the most common cause of the disease. *CAPN3* (LGMD2??) and *FKRP*(LGMD2??) were two other commonly mutated genes in our study (Table 2).

**Table 1:** Summary of findings in positive cases and mutation classification based on ACMG/AMP guidelines

Index ID	Gene	Mutation	Novel	Effect	Evidence of pathogenicity
10R-00963 10R-00534	*CAPN3*	NM_000070:c.801+1G>A	No	Pathogenic	PVS1, PP1-S, PM2, PM3, PP1-M, PP1, PP3, PP4
10R-00309 10R-00658 11R-02841 11R-03100	*FKRP*	NM_001039885:c.941C>T;p.T314M	No	Pathogenic	PP1-S, PM2, PM3, PP1-M, PP1, PP3, PP2, PP4
11R-00685	*ALG2*	NM_033087.3:indel:c.214_224delGGGGACTGGCT delinsAGTCCCCG;p.72_75delGDWLinsSPR	No	Pathogenic	PVS1, PP1-S, PM2, PM3, PM4, PP1, PP3, PP4
10R-00500 10R-00405 10R-00538 14R-02300 11R-01506 12R-01190	*DYSF*	NM_003494:c.164_165insA;p.I57Hfs*8	No	Pathogenic	PVS1, PP1-S, PM2, PM3, PM4, PP1-M, PP1, PP3, PP4
11R-00680 10R-00857	*SGCA*	NM_001135697:c.608_609insCG;p.S203fs	Yes	Pathogenic	PVS1, PP1-S, PM2, PM3, PM4, PP1-M, PP1, PP3, PP4
794310 502098 11R-00463	*SGCA*	NM_001135697:c.101G>A;p.R34H	No	Pathogenic	PM2, PP1-S, PM3, PP1-M, PP1, PP2, PP3, PP4
10R-00739	*CAPN3*	NM_000070:cA2329A>G;p.I777V	Yes	Pathogenic	PP1-S, PM2, PM3, PP1-M, PP1, PP2, PP3, PP4
10R-00751	*SEPN1*	NM_206926:c.1270G>A;p.D424N	Yes	Pathogenic	PP1-S, PM2, PM3, PP1-M, PP1, PP2, PP3, PP4
10R-00779	*CAPN3*	NM_000070:c.146G>A;p.R49H	No	Pathogenic	PP1-S, PM2, PM3, PP1-M, PP1, PP2, PP3, PP4
10R-00926	*SGCB*	NM_000232:c.355A>T;p.I119F	No	Pathogenic	PP1-S, PM2, PM3, PP1-M, PP1, PP2, PP3, PP4
10R-00973	*FKTN*	NM_006731:c.314G>T;p.C105F	No	Pathogenic	PP1-S, PM2, PM3, PP1-M, PP1, PP2, PP3, PP4
11R-00018 13R-01080	*CAPN3*	NM_000070:c.1699G>A;p.G567R	Yes	Likely pathogenic	PM2, PM3, PP2, PP3, PP4
11R-00031	*GNE*	NM_001190384:c.1805T>C;p.M602T	No	Pathogenic	PP1-S, PM2, PM3, PP1-M, PP1, PP2, PP3, PP4
11R-00230 12R-01188	*FKRP*	NM_001039885:c.941C>T;p.T314M	No	Pathogenic	PP1-S, PM2, PM3, PP1-M, PP1, PP2, PP3, PP4
11R-00232	*WNK1*	NM_213655:c.2152C>T:p.R718C	Yes	Pathogenic	PP1-S, PM2, PM3, PP1-M, PP1, PP2, PP3, PP4
11R-00308 12R-01189	*DYSF*	NM_001130976:c.1433delC;p.T478fs	Yes	Pathogenic	PVS1, PP1-S, PM2, PM3, PM4, PP1-M, PP1, PP4, PP3
11R-00337	*FKRP*	NM_001039885:c.1012G>T;p.V338L	Yes	Pathogenic	PP1-S, PM2, PM3, PP1-M, PP1, PP2, PP3, PP4
11R-00745	*DYSF*	NM_001130976:c.A5201T;p.E1734V	No	Pathogenic	PP1-S, PM2, PM3, PP1-M, PP1, PP2, PP3, PP4
11R-02618	*CAPN3*	NM_000070:c.310G>T;p.E104X	Yes	Pathogenic	PVS1, PM2, PM3, PM4, PP3, PP4
12R-00001	*ANO5*	NM_001142649, c.169C>T;p.R57W	No	Pathogenic	PP1-S, PM2, PM3, PP1-M, PP1, PP2, PP3, PP4
12R-00316	*CAPN3*	NM_000070:c.2381-1G>A	Yes	Pathogenic	PVS1, PM2, PM3, PM4, PP1-M, PP1-S, PP1, PP3, PP4
13R-01177	*DYSF*	NM_001130976:c.89-1G>A	Yes	Pathogenic	PVS1, PP1-S, PM2, PM3, PM4, PP1-M, PP1, PP3, PP4
14R-00183	*DNAJB6*	NM_005494:c.C287T;p.P96L	Yes	Pathogenic	PP1-S, PM2, PP1-M, PP1, PP2, PP3, PP4

*ACMG/AMP* American College of Medical Genetics and Genomics/Association for Molecular Pathology, *PVS1* pathogenic very strong, *PM2-4* pathogenic moderate, *PP1-4* pathogenic supporting, *PP1-M* pathogenic supporting (moderate), *PP1-S* pathogenic supporting (strong)

**Table 2:** Overview of the genetic diagnosis based on the neurological panel results

Genetic diagnosis	Number of families (*n* = 50)	Inheritance
Dyspherlinopathy	10 (20 %)	AR
Calpainopathy	8 (16 %)	AR
Dystroglycenopathy (type C)	7 (14 %)	AR
α-Sarcoglyconapthy	5 (10 %)	AR
β-Sarcoglyconapthy	1 (2 %)	AR
Dystroglycenopathy (type B)	1 (2 %)	AR
Anoctaminopathy	1 (2 %)	AR
Congenital disorder of glycosylation	1 (2 %)	AR
Rigid spine muscular dystrophy	1 (2 %)	AR
Nonaka myopathy	1 (2 %)	AR
Neuropathy	1 (2 %)	AR
Limb-girdle muscular dystrophy, type 1E	1 (2 %)	AD
Undiagnosed	12 (24 %)	

*AR* autosomal recessive, *AD* autosomal dominant

Three affected members of family 25 had a homozygous mutation in *FKRP* (c.C941T, p.T314M) that segregated with disease with the exception of a 3-year-old asymptomatic sister also homozygous for this allele. She probably has not developed the phenotype yet as the first symptoms associated with LGMD2I usually occur between the first and third decade of life.

In several families, mutations in genes related to non-LGMD myopathies were identified. Family 3 had a mutation in *ALG2* associated with a congenital disorder of glycosylation. The patients presented with a congenital limb-girdle pattern of weakness with no ocular or bulbar involvement. Muscle biopsies showed myopathic features, ragged red fibers, and a sub-sarcolemmal accumulation of structurally normal mitochondria. Family 16 had a mutation in *SEPN1* associated with rigid spine muscular dystrophy 1. The proband displayed major weakness in lower extremities that progressed and affected the upper proximal shoulder and girdle muscles. He suffered from respiratory failure and kyphoscoliosis. Needle EMG examination and nerve conduction studies were consistent with myopathy.

The index patient from family 24 presented with proximal muscle weakness of the lower extremities that started after the delivery of her first child. The condition progressed in severity and she started to note frequent falls and tripping after delivery of her second child. Nerve conduction studies as well as the needle EMG were consistent with a clinical diagnosis of progressive distal myopathy. In this patient, a mutation in *GNE* (UDP-*N*-acetyloglucosamine 2-epimerase/*N*-acetylmannosamine kinase) associated with inclusion body myopathy2/Nonaka myopathy was identified. Neuropathy due to mutation in *WNK1* was found to underlie disease in family 26. The female patient was normal until the age of 9, when she had a problem with standing and walking. She developed full Gower’s sign and had frequent falls. The girl had a global proximal weakness affecting, lower more than the upper, extremities. There was also bilateral mild hypertrophy of the calf muscles with a marked lordotic gait. Muscle biopsy showed clear dystrophic changes. She had a similarly affected sister.

We performed WES on nine neurological panel “negative” consanguineous multiplex families with autosomal recessive disease inheritance. Accordingly, we concentrated on finding pathogenic homozygous variants within defined ROHs (shared by affected individuals only). WES did not reveal any such variants, in particular among neuromuscular genes within these regions.

## Discussion

High-throughput solutions such as NGS have revolutionized the genetic approach for molecular diagnosis of Mendelian disorders. Utility of WES in the clinic has been relatively successful, mainly for neurological conditions, with a yield of ~25 % [9, 13]. Due to high cost, a long turnaround time and challenging interpretation, arguably, WES should be considered mainly for the purpose of novel gene discovery. An alternative solution that substantially reduces the above limitations is an approach which targets known disease genes grouped in panels [14]. Several approaches have been taken to design and sequence NGS-based gene panels for LGMD patients [7, 15–19]. In our study, the neurological gene panel comprised 759 genes known to cause Mendelian neurological diseases (not only LGMD) as annotated by OMIM up to August 2013. The neurological panel is a part of our Mendeliome assay that includes 13 symptom/sign-based gene panels [14]. Minimal expertise is required by clinicians to choose the neurological panel from the 13 present in the “Mendeliome,” e.g., any muscle weakness or movement problem will trigger testing for this panel. This comprehensive assay simplifies the molecular diagnostic process and takes into consideration the remarkable phenotypic variability between many neuromuscular disorders. In this way, we significantly reduce the chance of missing a genetic diagnosis due to atypical presentation.

In our cohort of 50 LGMD families, the neurological panel was able to resolve 76 % of cases (38/50 families), an exceptionally high diagnostic yield relative to previously published data (Table 3). In light of ambiguity associated with clinical significance of NGS variants, assigning an objective assessment of pathogenicity is crucial in making a molecular diagnosis. We interpreted the clinical significance of our findings (those which survived our filtration process) using ACMG/AMP standards and guidelines. This objective and standardized approach to variant classification, while clearly indicated in clinical situations, has not been applied by previous studies evaluating NGS for diagnosis of myopathies [7, 15–21]. Given the nature and number of variants identified using NGS, we feel that classification in this manner is not only useful but also essential to clear interpretation by referring physicians. All but one disease associated variant identified in our cohort were classified as pathogenic. The one exception was classified as a likely pathogenic, consequent to there being only one affected member in the family, thus preventing demonstration of co-segregation with multiple affected individuals (Table 1).

**Table 3:** Diagnostic yield of gene panels in LGMD/myopathic patients

Gene Panel	Number of genes in the gene panel	Number of families studied	Number of genetically diagnosed families
Ghosh at al. [15]	9	27	10 (37 %)
Savarese et al. [7]	93	177	108 (61 %)
Seong et al. [17]	18	35	20 (57 %)
Ankala et al. [18]	11	96	25 (26 %)
Dai et al. [19]	399	55	36 (65 %)
Kuhn et al. [20]	38	58	19 (33 %)
Our study	759	50	38 (76 %)

The panel covers all types of muscular dystrophies, myopathies, and other neuromuscular disorders encompassing more than 300 diseases to date [22]. Unfortunately, most of them have very similar clinical presentations and even with thorough clinical evaluation and muscle pathology, a correct diagnosis without genetic testing remains difficult. A strong case may be made to test all muscle genes in analysis of patients with suspected LGMD [20]. In similar studies, a diagnostic yield of LGMD patients using a gene panel approach varies from 16 to 65 % [7, 20]. In these studies, the number and composition of genes sequenced were associated with diagnostic yield. Ankala and colleagues, by expanding their LGMD panel with 11 genes to a more comprehensive neuromuscular disease (NMD) panel containing 41 genes, achieved a threefold greater diagnostic rate. Their NMD panel also covered other non-LGMD movement disorders and increased the yield from approximately 15 to 46 % [18]. Dai et al. claimed to have designed a panel of 44 known genes underlying muscular dystrophies and congenital myopathies. In fact, their libraries incorporated 399 genes covering common inherited disorders including at least 55 genes associated with myopathies. They were able to find causative mutations in 65 % of patients [19]. The MotorPlex assay comprising 93 muscle disease loci identified pathogenic or potential causative variants in 61 % of patients tested [7]. Better performance of our panel (diagnostic yield = 76 %) versus others described may be associated with it being more comprehensive (759 genes) and its application in an inbred population. Our neurological panel was able to diagnose patients due to mutations in genes absent in assays described in other studies. Those gene panels would miss at least 8 to 10 % of pathogenic changes in our cohort. The Athena Diagnostics LGMD gene panel would not detect a mutation in seven of our cases: *FKTN*, *ANO5*, *DNAJB6*, *SEPN1*, *GNE*, *WNK1*, and *ALG2* [15]. Both assays used by Ankala et al. and Seong et al. would miss diagnosis due to changes in four to five genes (*DNAJB6*, *GNE*, *WNK1*, *ALG2*, and *SEPN1*) [17, 18]. Two other panels would fail to diagnose patients from our cohort with disorders associated with *DNAJB6*, *WNK1*, and *ALG2* [19] and *SEPN1*, *WNK1*, and *ALG2* [20]. While our neurological panel could be focused further, there is little to be gained from doing so other than to perhaps reduce incidental findings. Ghaoui and colleagues tested 60 LGMD families (undiagnosed by conventional candidate gene sequencing) using WES with a diagnostic success rate of 45 %. The identified mutations were present only in known myopathy genes and WES did not reveal any finding in novel genes. The group obtained identical results by retesting the samples using a neuromuscular panel which contained 336 neuromuscular disease-related genes. The panel confirmed all variants identified by WES highlighting the benefit of a panel-based approach relative to candidate gene sequencing or WES [21]. Comprehensive gene panels should be viewed as first-tier tests before considering WES. Based on our results and reports in the literature cited above, we consider application of gene panels to be a more effective approach for diagnosis of myopathies.

Considering very high genetic heterogeneity (similar phenotype associated with multiple genes) and phenotypic heterogeneity (a single gene associated with multiple phenotypes) in LGMD and other myopathies, applying a gene panel (such as our neurological panel), incorporating a comprehensive list of genes associated with neuromuscular disorders, that can be tested together at the same time, provides a very powerful and practical diagnostic tool. Besides the high diagnostic efficiency, our neurological panel is cost-effective. A multiplexing strategy running 24 samples per run dramatically reduces the sample processing cost (~$150) and time. A similar approach was taken by Savarese and colleagues who designed the MotorPlex panel (93 genes) covering all known forms of non-syndromic muscle disorders. They also applied a cost-effective pooled sequencing strategy with 100 % specificity and sensitivity of the assay in 20 LGMD or congenital myopathy patients [7].

Molecular diagnosis is crucial for genetic counseling and prognosis [23, 24]. An earlier genetic diagnosis provides better disease management and also protects patients from more invasive clinical evaluation [25].

WES applied to the remaining undiagnosed multiplex families (*n* = 9) did not detect any disease-causing mutations. The use of NGS technology, as applied in this study, has limitations including incomplete coverage of target sequences in PCR based libraries, amplification bias resulting non-uniform coverage of library amplicons, inability to detect structural changes, and poor sensitivity for copy number variation. We cannot exclude possibility of missing mutations in known genes which are not fully covered or those present in intronic regions which are not covered by WES [26, 27]. Another NGS limitation is copy number variation (CNV) (gross deletions and insertions) which are poorly detected so far by this approach [20]. WES results of our and other studies [21] also suggest that the majority of genes underlying LGMD and other myopathies have probably been identified with limited scope for novel discovery.

## Conclusions

We have demonstrated that our neurological panel assay covering 759 neurological genes cited by OMIM has a high diagnostic yield (76 %) for LGMD and other myopathies. In addition, it is a rapid and cost-effective assay. We believe that the majority of LGMD patients can be diagnosed using this new very powerful genomic tool and WES should be reserved only for negative cases as an opportunity to discover novel candidate genes. Classification of disease-associated variants with respect to pathogenicity, using guidelines of the ACMG/AMP as applied in our study further, adds to the power and utility of NGS panels for the clinical diagnosis of LGMD and other myopathies.

## Methods

### Participants

A total of 50 random genetically unstudied families were included in this study; 36 and 14 of these were multiplex and simplex families, respectively. All were collected through neurosciences clinic at KFSHRC between 2010 and 2015. The age of disease onset varied from 1 to 35 years. All patients presented with muscle weakness affecting the pelvic and shoulder girdle. Muscle biopsy in all individuals showed dystrophic or myopathic changes. DNA was extracted from peripheral blood samples using standard procedures (Flexi Gene DNA Handbook, Qiagen). Samples were quantitated spectrophotometrically and stored at −20 °C.

### Neurological panel assay and bioinformatics analysis

This gene panel was a part of the NGS targeted resequencing “Mendeliome” assay that consists of 13 symptom-based gene panels which cover all inherited disease associated genes in OMIM as of August 2013 [14]. The neurological panel included 759 OMIM genes associated with neurological disorders (see Additional file 2). Genes were amplified and a library constructed using an AmpliSeq HiFi mix, proprietary primers (see Additional file 3) and library kit (Thermo Fisher, Carlsband, CA, USA) followed by sequencing on the Ion Proton platform according to the manufacturer’s protocol (Thermo Fisher, Carlsband, CA, USA). Variants were called and annotated using the Saudi Human Genome pipeline [14]. Briefly, only regions of the reads with high quality (Ion Torrent base calling algorithm, Thermo Fisher, Carlsband, CA, USA) were aligned to the UCSC hg19 (http://genome.ucsc.edu/) reference sequence and processed for variant calling using the Torrent Suite Variant Caller (TVC) program (Thermo Fisher, Carlsband, CA, USA). Performance of the neurological panel in this study resulted in >95 % of reads at Q17 with an average read depth of 166 X. Variants were annotated using in-house programs that extend the public Annovar package with other licensed commercial data sets such as the professional version of HGMD [28] and in-house databases made up of a collection of disease-causing and polymorphic variants observed in individuals of Arab ethnicity. As a final step, non-relevant variants were filtered out based on their quality, functional characteristics, and their frequency in our datasets. Intronic variants, synonymous variants, and those present in population databases (specifically those that are in 1000 Genome database with MAF >1 %) were also filtered out. Furthermore, variants that were frequent (MAF >1 %) in our population specific in-house variant database were also filtered out. After applying filtration criteria, all nonsense, frameshift, and canonical splice site variants were considered pathogenic. For interpretation and classification of the remaining SNVs, nucleotide and amino acid conservation and effects on protein sequence were analyzed with PolyPhen-2 (http://genetics.bwh.harvard.edu/pph2/) and SIFT (http://sift.jcvi.org/). Potential causative variants were validated by Sanger sequencing and further vetted for familial segregation. Finally, the remaining variants were identified as pathogenic or likely pathogenic following guidelines of the American College of Medical Genetics (ACMG) and Association for Molecular Pathology (AMP) [29].

### DNA Sanger sequencing

Coding regions of candidate genes were sequenced using a BigDye Terminator kit (Applied Biosystems, Foster City, CA) and run on an ABI 3730xl automated sequencer (Applied Biosystems, Foster City, CA). SeqScape v.2.6 software (Applied Biosystems, Foster City, CA) was used to align sequence data against the relevant reference.

### Genetic variant interpretation, ACMG/AMP guidelines

The clinical significance of NGS variants was classified using an openly available online tool for implementing the ACMG/AMP standards and guidelines: http://medschool.umaryland.edu/Genetic_Variant_Interpretation_Tool1.html. [30].

### Genotyping and homozygosity mapping

All participating individuals (affected and unaffected) in cases where the neurological panel failed to identify a likely casual mutation were genotyped using the Affymetrix Axiom array (Affymetrix, Santa Clara, CA, USA) following the manufacturer’s protocol (http://www.affymetrix.com/support/technical/manuals.affx). Resulting genotypes were analyzed for shared runs of homozygosity (ROH) using autoSNPa (http://dna.leeds.ac.uk/autosnpa/).

### Whole exome sequencing and analysis

Multiplex families that tested negative on the neurological panel underwent WES. Briefly, 100 ng of each DNA was amplified in 12 separate wells using Exome Primer Pools, AmpliSeq HiFi mix (Thermo Fisher, Carlsbad, CA, USA) and 10 amplification cycles. All 12 PCR pools were combined in one well and subjected to primer digestion by incubation with FuPa reagent (Thermo Fisher, Carlsbad, CA, USA). Amplified Exome targets were ligated with Ion P1 and Ion Xpress Barcode adapters. After purification, libraries were quantified using qPCR with the Ion Library Quantification Kit (Thermo Fisher, Carlsbad, CA, USA). The prepared exome library was further used for emulsion PCR on an Ion OneTouch System and templated Ion Sphere particles were enriched using Ion OneTouch ES, both procedures following the manufacturer’s instructions (Thermo Fisher, Carlsbad, CA, USA). The template-positive Ion PI Ion Sphere particles were processed for sequencing on the Ion Proton instrument (Thermo Fisher, Carlsbad, CA, USA). Reads were mapped to UCSC hg19 (http://genome.ucsc.edu/) and variants identified using the Saudi Human Genome Program (SHGP) pipeline [14].

## Additional files

Additional file 1: Clinical characteristics and biopsy results of index cases for families studied. (DOCX 223 kb) Additional file 2: Genes included in the neurological panel. (DOC 97 kb) Additional file 3: Amplicon details and primer sequences for the neurological panel. (XLSX 1256 kb)

## References

[1] Khadilkar SV, Singh RK (2008). Limb girdle muscular dystrophies in India. Neurol India.

[2] Laing NG (2012). Genetics of neuromuscular disorders. Crit Rev Clin Lab Sci.

[3] Murphy A, Kelly RJ (2015). From molecular classification to targeted therapeutics: the changing face of systemic therapy in metastatic gastroesophageal cancer. Gastroenterol Res Pract.

[4] Nigro V, Savarese M (2014). Genetic basis of limb-girdle muscular dystrophies: the 2014 update. Acta Myol.

[5] Narayanaswami P, Weiss M, Selcen D, David W, Raynor E, Carter G, Wicklund M, Barohn RJ, Ensrud E, Griggs RC (2014). Evidence-based guideline summary: diagnosis and treatment of limb-girdle and distal dystrophies: report of the guideline development subcommittee of the American Academy of Neurology and the practice issues review panel of the American Association of Neuromuscular & Electrodiagnostic Medicine. Neurology.

[6] Guglieri M, Straub V, Bushby K, Lochmuller H (2008). Limb-girdle muscular dystrophies. Curr Opin Neurol.

[7] Savarese M, Di Fruscio G, Mutarelli M, Torella A, Magri F, Santorelli FM, Comi GP, Bruno C, Nigro V (2014). MotorPlex provides accurate variant detection across large muscle genes both in single myopathic patients and in pools of DNA samples. Acta Neuropathol Commun.

[8] Fogel BL, Lee H, Deignan JL, Strom SP, Kantarci S, Wang X, Quintero-Rivera F, Vilain E, Grody WW, Perlman S (2014). Exome sequencing in the clinical diagnosis of sporadic or familial cerebellar ataxia. JAMA Neurol.

[9] Lee H, Deignan JL, Dorrani N, Strom SP, Kantarci S, Quintero-Rivera F, Das K, Toy T, Harry B, Yourshaw M (2014). Clinical exome sequencing for genetic identification of rare Mendelian disorders. JAMA.

[10] Vasli N, Bohm J, Le Gras S, Muller J, Pizot C, Jost B, Echaniz-Laguna A, Laugel V, Tranchant C, Bernard R (2012). Next generation sequencing for molecular diagnosis of neuromuscular diseases. Acta Neuropathol.

[11] Alkuraya FS (2014). Genetics and genomic medicine in Saudi Arabia. Mol Genet Genomic Med.

[12] Abu-Safieh L, Alrashed M, Anazi S, Alkuraya H, Khan AO, Al-Owain M, Al-Zahrani J, Al-Abdi L, Hashem M, Al-Tarimi S (2013). Autozygome-guided exome sequencing in retinal dystrophy patients reveals pathogenetic mutations and novel candidate disease genes. Genome Res.

[13] Yang Y, Muzny DM, Reid JG, Bainbridge MN, Willis A, Ward PA, Braxton A, Beuten J, Xia F, Niu Z (2013). Clinical whole-exome sequencing for the diagnosis of mendelian disorders. N Engl J Med.

[14] Saudi Mendeliome G (2015). Comprehensive gene panels provide advantages over clinical exome sequencing for Mendelian diseases. Genome Biol.

[15] Ghosh PS, Zhou L (2012). The diagnostic utility of a commercial limb-girdle muscular dystrophy gene test panel. J Clin Neuromuscul Dis.

[16] Bartoli M, Desvignes JP, Nicolas L, Martin K (2014). Exome sequencing as a second-tier diagnostic approach for clinically suspected dysferlinopathy patients. Muscle Nerve.

[17] Seong MW, Cho A, Park HW, Seo SH, Lim BC, Seol D, Cho SI, Park SS, Chae JH: Clinical applications of next-generation sequencing-based gene panel in patients with muscular dystrophy: Korean experience. Clin Genet 2015. (Epub ahead of print). 10.1111/cge.12621 26060040

[18] Ankala A, da Silva C, Gualandi F, Ferlini A, Bean LJ, Collins C, Tanner AK, Hegde MR (2015). A comprehensive genomic approach for neuromuscular diseases gives a high diagnostic yield. Ann Neurol.

[19] Dai Y, Wei X, Zhao Y, Ren H, Lan Z, Yang Y, Chen L, Cui L (2015). A comprehensive genetic diagnosis of Chinese muscular dystrophy and congenital myopathy patients by targeted next-generation sequencing. Neuromuscul Disord.

[20] Kuhn M, Glaser D, Joshi PR, Zierz S, Wenninger S, Schoser B, Deschauer M (2016). Utility of a next-generation sequencing-based gene panel investigation in German patients with genetically unclassified limb-girdle muscular dystrophy. J Neurol.

[21] Ghaoui R, Cooper ST, Lek M, Jones K, Corbett A, Reddel SW, Needham M, Liang C, Waddell LB, Nicholson G, et al: Use of Whole-Exome Sequencing for Diagnosis of Limb-Girdle Muscular Dystrophy: Outcomes and Lessons Learned. JAMA Neurol. 2015;72:11424-1432. 10.1001/jamaneurol.2015.2274 26436962

[22] Kaplan JC, Hamroun D (2014). The 2015 version of the gene table of monogenic neuromuscular disorders (nuclear genome). Neuromuscul Disord.

[23] Leung DG, Wagner KR (2013). Therapeutic advances in muscular dystrophy. Ann Neurol.

[24] Mercuri E, Muntoni F (2013). Muscular dystrophies. Lancet.

[25] Vasli N, Laporte J (2013). Impacts of massively parallel sequencing for genetic diagnosis of neuromuscular disorders. Acta Neuropathol.

[26] Biesecker LG, Biesecker BB (2014). An approach to pediatric exome and genome sequencing. Curr Opin Pediatr.

[27] Biesecker LG, Green RC (2014). Diagnostic clinical genome and exome sequencing. N Engl J Med.

[28] Wang K, Li M, Hakonarson H (2010). ANNOVAR: functional annotation of genetic variants from high-throughput sequencing data. Nucleic Acids Res.

[29] Richards S, Aziz N, Bale S, Bick D, Das S, Gastier-Foster J, Grody WW, Hegde M, Lyon E, Spector E (2015). Standards and guidelines for the interpretation of sequence variants: a joint consensus recommendation of the American College of Medical Genetics and Genomics and the Association for Molecular Pathology. Genet Med.

[30] Kleinberger J, Maloney KA, Pollin TI, Jeng LJ: An openly available online tool for implementing the ACMG/AMP standards and guidelines for the interpretation of sequence variants. Genet Med 2016. (Epub ahead of print). 10.1038/gim.2016.13 PMC5026899 26986878

