# Supplementary material for: A first-line diagnostic assay for limb-girdle muscular dystrophy and other myopathies
Source: Hum Genomics. 2016 Sep 27;10:32. doi: 10.1186/s40246-016-0089-8 (PMC5037890; doi:10.1186/s40246-016-0089-8)
Supplement: Additional file 1: — Clinical characteristics and biopsy results of index cases for families studied. (DOCX 223 kb) [file 40246_2016_89_MOESM1_ESM.docx]

**Additional** **File1 - Genes included in the neurological panel**

| AAAS | CAPN3 | DRD2 | HINT1 | MT-ND3 | PHGDH | SIGMAR1 | TRPV4 |
| --- | --- | --- | --- | --- | --- | --- | --- |
| AARS | CASC5 | DRD4 | HIST1H4B | MT-TV | PHOX2B | SIL1 | TSC1 |
| AARS2 | CASK | DRD5 | HIST3H3 | MT-TW | PHYH | SIX3 | TSC2 |
| ABAT | CASP2 | DST | HK1 | MTFMT | PIGN | SLC12A6 | TSEN2 |
| ABCD1 | CAV3 | DYNC1H1 | HOXB1 | MTM1 | PIK3R2 | SLC1A3 | TSEN34 |
| ABHD12 | CC2D1A | DYRK1A | HOXD10 | MTMR2 | PIK3R5 | SLC20A2 | TSEN54 |
| ACAD9 | CCDC78 | DYSF | HSD17B10 | MTPAP | PINK1 | SLC25A19 | TSFM |
| ACO2 | CCDC88C | EARS2 | HSD17B4 | MUSK | PIP5K1C | SLC25A22 | TSPAN7 |
| ACSL4 | CCNA2 | EEF1B2 | HSPB1 | MYBPC1 | PLA2G6 | SLC2A1 | TTBK2 |
| ACTA1 | CCT5 | EFHC1 | HSPB3 | MYF6 | PLCB1 | SLC30A10 | TTC19 |
| ACTB | CDC6 | EGR2 | HSPB8 | MYH2 | PLEC | SLC31A1 | TTI2 |
| ACTB | CDH15 | EIF2B1 | HSPD1 | MYH3 | PLEKHG5 | SLC33A1 | TTN |
| ACTG1 | CDK5RAP2 | EIF2B2 | HTRA2 | MYH7 | PLP1 | SLC35A2 | TTPA |
| ACY1 | CDKL5 | EIF2B3 | HTT | MYH8 | PMM2 | SLC52A2 | TUBA1A |
| ADAR | CDON | EIF2B4 | HUWE1 | MYOT | PMP22 | SLC52A3 | TUBA8 |
| ADCK3 | CENPJ | EIF2B5 | HYLS1 | NAGA | PNKD | SLC5A7 | TUBB2B |
| ADCK3 | CEP135 | EIF4G1 | IAPP | NBN | PNKP | SLC6A3 | TUBB3 |
| ADK | CEP152 | ELP2 | IER3IP1 | NDE1 | PNPLA2 | SLC6A5 | TUBB4A |
| ADRA2B | CEP41 | EMD | IGBP1 | NDRG1 | PNPLA6 | SLC6A8 | TUBGCP6 |
| ADSL | CEP57 | EMX2 | IGHMBP2 | NDST1 | PNPO | SLC9A6 | TUFM |
| AFF2 | CEP63 | ENTPD1 | IKBKAP | NDUFA1 | POLG | SLITRK1 | TUSC3 |
| AFG3L2 | CFL2 | EPB41L1 | IL1RAPL1 | NDUFA10 | POLR3A | SMARCA2 | TYMP |
| AGRN | CHAT | EPM2A | INF2 | NDUFA11 | POLR3B | SMARCA4 | TYROBP |
| AGTR2 | CHKB | ERBB3 | INPP4A | NDUFA12 | POMGNT1 | SMARCB1 | UBA1 |
| AHI1 | CHMP1A | ERLIN2 | INPP5E | NDUFA2 | POMT1 | SMCHD1 | UBE2A |
| AIMP1 | CHMP2B | EXOSC3 | IQSEC2 | NDUFA7 | POMT2 | SMN1 | UBE3A |
| ALDH5A1 | CHRNA1 | FA2H | ISCU | NDUFA9 | PPP2R2B | SMS | UBQLN2 |
| ALDH7A1 | CHRNA2 | FAM126A | ISPD | NDUFAB1 | PPT1 | SNAP29 | UBR7 |
| ALS2 | CHRNA4 | FAM134B | ITGA7 | NDUFAF1 | PQBP1 | SNCA | UPF3B |
| AMPD1 | CHRNB1 | FARS2 | ITM2B | NDUFAF2 | PRICKLE1 | SNCB | UQCRB |
| ANG | CHRNB2 | FASN | ITPR1 | NDUFAF3 | PRICKLE2 | SNIP1 | UQCRC2 |
| ANO10 | CHRND | FASTKD2 | JPH3 | NDUFAF4 | PRKCG | SOBP | UQCRQ |
| ANO3 | CHRNE | FBXO7 | KANK1 | NDUFAF5 | PRKRA | SOD1 | UROC1 |
| ANO5 | CHRNG | FGD1 | KARS | NDUFAF6 | PRMT10 | SPAST | VANGL1 |
| AP1S2 | CLCN1 | FGD4 | KBTBD13 | NDUFB3 | PRNP | SPG11 | VAPB |
| AP4B1 | CLCN2 | FGF14 | KCNA1 | NDUFS1 | PRPS1 | SPG20 | VAPB |
| AP4E1 | CLN3 | FHL1 | KCNC3 | NDUFS2 | PRRT2 | SPG21 | VCP |
| AP4M1 | CLN5 | FIG4 | KCNJ10 | NDUFS3 | PRSS12 | SPG7 | VLDLR |
| AP4S1 | CLN6 | FKRP | KCNMA1 | NDUFS4 | PRX | SPR | VMA21 |
| AP5Z1 | CLN8 | FKTN | KCNQ2 | NDUFS5 | PSAP | SPTAN1 | VPS13A |
| APOB | CMT1A | FLNA | KCNQ3 | NDUFS6 | PSEN1 | SPTBN2 | VPS33B |
| APTX | CNBP | FLNC | KCNT1 | NDUFS7 | PSEN2 | SPTLC1 | VPS35 |
| AR | CNKSR1 | FLVCR2 | KCTD7 | NDUFS8 | PTCH1 | SPTLC2 | VPS37A |
| ARFGEF2 | CNTN1 | FMR1 | KDM5A | NDUFV1 | PTEN | SRPX2 | VRK1 |
| ARHGEF6 | CNTNAP2 | FOLR1 | KDM5C | NDUFV2 | RAB18 | ST3GAL3 | WDR45 |
| ARHGEF9 | COA5 | FOXG1 | KDM6B | NEB | RAB39B | ST3GAL5 | WDR45B |
| ARID1A | COG6 | FOXP1 | KIAA0196 | NEFL | RAB7A | STAMBP | WDR62 |
| ARID1B | COL4A2 | FOXP2 | KIF1A | NFU1 | RABL6 | STIL | WDR81 |
| ARL13B | COL6A1 | FOXRED1 | KIF1B | NGF | RAD50 | STIM1 | WNK1 |
| ARL14EP | COL6A2 | FRY | KIF5A | MT-ND4 | RAI1 | STRADA | YARS |
| ARNT2 | COL6A3 | FTL | KIF7 | NHLRC1 | RALGDS | STXBP1 | ZBTB40 |
| ARSA | COLQ | FTSJ1 | KIRREL3 | NIN | RAPSN | SUCLG1 | ZCCHC8 |
| ARX | COQ2 | FUS | L1CAM | NIPA1 | RARS2 | SUOX | ZDHHC15 |
| ASCC3 | COQ2 | FUS | LAMA1 | NKX2-1 | RBBP8 | SURF1 | ZDHHC9 |
| ASCL1 | COQ5 | FUZ | LAMA2 | NLGN3 | REEP1 | SYN1 | ZEB2 |
| ASPM | COQ6 | FXN | LAMB1 | NLGN4X | RELN | SYNE1 | ZFYVE26 |
| ATCAY | COX10 | GABRA1 | LAMC3 | NOL3 | RGS7 | SYNE2 | ZFYVE27 |
| ATL1 | COX14 | GABRB3 | LARGE | NOP56 | RMND1 | SYNGAP1 | ZIC2 |
| ATN1 | COX15 | GABRD | LDB3 | NPC1 | RNASEH2A | SYP | ZNF335 |
| ATP13A2 | COX6B1 | GABRG2 | LGI1 | NPC2 | RNASEH2B | SYT14 | ZNF41 |
| ATP1A2 | CP | GAD1 | LINS | NRXN1 | RNASEH2C | TACO1 | ZNF526 |
| ATP1A3 | CPA6 | GALC | LITAF | NTRK1 | RNASET2 | TAF1 | ZNF592 |
| ATP2A1 | CRADD | GAMT | LMNA | NUBPL | RNF170 | TAF2 | ZNF674 |
| ATP2B3 | CRBN | GAN | LMNB1 | NUP62 | RNU4ATAC | TARDBP | ZNF711 |
| ATP5E | CRYAB | GARS | LRPPRC | OCLN | ROGDI | TBC1D24 | ZNF81 |
| ATP6AP2 | CSF1R | GATAD2B | LRRK2 | OCRL | RPGRIP1L | TBP |  |
| ATP7A | CSNK1D | GATM | LRSAM1 | OPHN1 | RPS6KA3 | TCAP |  |
| ATPAF2 | CSTB | GBA2 | MAGT1 | OPTN | RTN2 | TCTN1 |  |
| ATR | CTNNB1 | GDAP1 | MAN1B1 | ORC1 | RTTN | TDGF1 |  |
| ATRX | CTSD | GDI1 | MAOA | ORC6 | RYR1 | TDP1 |  |
| ATXN1 | CUL4B | GFAP | MAPK10 | PABPN1 | SACS | TECPR2 |  |
| ATXN10 | CYP27A1 | GFER | MAPT | PACS1 | SAMHD1 | TECR |  |
| ATXN2 | CYP2U1 | GFPT1 | MATR3 | PAFAH1B1 | SBF2 | TFG |  |
| ATXN3 | CYP7B1 | GIGYF2 | MBD5 | PAK3 | SC5D | TGIF1 |  |
| ATXN7 | D4Z4 | GJB1 | MCPH1 | PANK2 | SCAPER | TGM6 |  |
| ATXN8 | DAG1 | GJC2 | MECP2 | PARK2 | SCARB2 | TH |  |
| ATXN8OS | DARS2 | GLI2 | MED12 | PARK7 | SCN1A | THAP1 |  |
| AUTS2 | DCC | GLRA1 | MED13L | PARP1 | SCN1B | TIA1 |  |
| B3GALNT2 | DCTN1 | GLRB | MED17 | PC | SCN2A | TK2 |  |
| BAG3 | DCTN1 | GNAL | MED23 | PCDH19 | SCN4A | TMEM135 |  |
| BCS1L | DCX | GNB4 | MED25 | PDGFRB | SCN8A | TMEM138 |  |
| BDMR | DDHD1 | GNE | MEF2C | PDHA1 | SCN9A | TMEM216 |  |
| BDNF | DDHD2 | GON4L | MEGF10 | PDHB | SCO1 | TMEM237 |  |
| BIN1 | DES | GOSR2 | MFF | PDHX | SCO2 | TMEM5 |  |
| BRAT1 | DHTKD1 | GPHN | MFSD8 | PDSS2 | SDHA | TMEM67 |  |
| BRWD3 | DIP2B | GPR56 | MLC1 | PDX1 | SDHAF1 | TMEM70 |  |
| BSCL2 | DIS3L2 | GPR98 | MMD2 | PDYN | SEPN1 | TNNI2 |  |
| BUB1B | DLG3 | GRIA3 | MOG | PECR | SEPSECS | TNNT1 |  |
| C12orf57 | DMD | GRIK2 | MPC1 | PEX1 | SERPINI1 | TNNT3 |  |
| C12orf65 | DMPK | GRIN1 | MPDZ | PEX12 | SETX | TOR1A |  |
| C19orf12 | DNA2 | GRIN2A | MPP3 | PEX14 | SGCA | TPK1 |  |
| C5orf42 | DNAJB2 | GRIN2B | MPZ | PEX16 | SGCB | TPM2 |  |
| C9orf72 | DNAJB6 | GRM1 | MR1 | PEX2 | SGCD | TPM3 |  |
| CA8 | DNAJC5 | GRN | MRE11A | PEX26 | SGCE | TPP1 |  |
| CACNA1A | DNM2 | GTDC2 | MRI1 | PEX3 | SGCG | TRAPPC9 |  |
| CACNA1G | DNMT1 | HCFC1 | MRPL3 | PEX5 | SH3TC2 | TREM2 |  |
| CACNB4 | DOK7 | HDAC1 | MSTN | PEX6 | SHANK2 | TREX1 |  |
| CACNG2 | DPAGT1 | HDAC4 | MT-ATP6 | PFN1 | SHANK3 | TRIM32 |  |
| CAMTA1 | DPM2 | HEPACAM | MT-ATP8 | PHC1 | SHH | TRMT1 |  |
| CAPN10 | DPYD | HEXA | MT-CO2 | PHF8 | SHROOM4 | TRPA1 |  |
