# Supplementary material for: A first-line diagnostic assay for limb-girdle muscular dystrophy and other myopathies
Source: Hum Genomics. 2016 Sep 27;10:32. doi: 10.1186/s40246-016-0089-8 (PMC5037890; doi:10.1186/s40246-016-0089-8)
Supplement: Additional file 2: — Genes included in the neurological panel. (DOC 97 kb) [file 40246_2016_89_MOESM2_ESM.doc]

**Additional File 2. Clinical characteristics and biopsy results of index cases for families studied.**

| Family | Index ID | Familial/ Sporadic | Age of Onset | Distribution of Weakness | Other Clinical Characteristics | CK (U/L) | Biopsy Findings |
| --- | --- | --- | --- | --- | --- | --- | --- |
| 1 | 10R-00963 | Familial | 17 | LGMW | Weak hip adductors | 4950 | Muscular dystrophy, absent γ-sarcoglycan on IHC |
| 2 | 10R-00309 | Sporadic | 2 | LGMW | Hypertrophic calves; steroid responsive | 2951 | Muscular dystrophy, partial loss of δ-sarcoglycan on IHC |
| 3 | 10R-00484 | Familial | 6 | LGMW | Fatigability | 258 | Myopathic changes with marked mitochondrial proliferation, ragged red fibers and a sub-sarcolemmal accumulation of structurally normal mitochondria |
| 4 | 10R-00500 | Familial | 14 | LGMW + Distal (Mixed) | Posterior leg muscle involvement | 5000 | Muscular dystrophy, marked reduction of all sarcoglycans on IHC |
| 6 | 10R-00359 | Sporadic | 6 | Distal > Proximal, Post >Proximal | MRI posterior legs and thigh atrophy | 1201 | Muscular dystrophy with mild mitochondrial proliferation, mild reduction of alpha and δ-sarcoglycan on IHC |
| 7 | 10R-00405 | Sporadic | 15 | LGMW, Pelvic girdle weakness | Dystrophic; myopathic | 1580 | Muscular dystrophy with mild mitochondrial proliferation, normal IHC |
| 8 | 11R-00680 | Familial | 3 | LGMW | Contracture wheel chaired at 14 yrs | 2800 | Muscular dystrophy |
| 10 | 10R-00538 | Familial | 16 | Distal postural and LGMW (Mixed) | MRI posterior atrophy of calf and leg muscles | 9500 | Necrotizing myopathy with mild mitochondrial proliferation, absent dysferlin on IHC |
| 11 | 794310 MC | Sporadic | 4 | LGMW | Contracture at Achilles tendons | 4400 | Myopathic changes, absent all sarcoglycans on IHC |
| 12 | 10R-00534 | Familial | 14 | LGMW, Distal more than proximal | - | 8500 | Muscular dystrophy, normal IHC |
| 13 | 502098 MC | Familial | 3 | LGMW | Contracture; bedridden at 14 yrs; abnormal EKG | 2550 | Muscular dystrophy, normal IHC |
| 14 | 10R-00658 | Familial | 1 | LGMW (V) | Steroid responsive; respiratory failure | 6380 | Muscular dystrophy, normal IHC |
| 15 | 10R-00739 | Familial | 18 | Distal (MM) | Distal posterior limb muscle atrophy and weakness | 2508 | Muscular dystrophy with mild mitochondrial proliferation and Type 2 predominance, absent dysferlin on IHC |
| 16 | 10R-00751 | Familial | 5 | LGMW and Paraspinal muscle weakness | Lordosis; respiratory failure | 182 | Dystrophic muscle with minicores and type 1 predominence, normal IHC |
| 17 | 10R-00779 | Familial | 8 | LGMW | Wheel chaired at 16 yrs | 1446 | Muscular dystrophy with mild inflammation, moderate reduction of all sarcoglycans except α on IHC |
| 18 | 10R-00857 | Sporadic | 9 | LGMW, Legs weaker than legs | Wheel chaired at 18 yrs | 200 | Muscular dystrophy with mild mitochondrial proliferation, normal IHC |
| 21 | 10R-00926 | Familial | 15 | LGMW | Severe clinical course; wheel chaired at 12 yrs | 10000 | Muscular dystrophy, absent γ and reduction of other sarcoglycans |
| 22 | 10R-00973 | Familial | 2 | Severe and Generalized (Global) | Congenital hypotonia | 1905 | No dystrophic changes. Partial merosin deficiency |
| 23 | 11R-00018 | Familial | 5 | LGMW | Contracture knees and Achilles | 3500 | Muscular dystrophy, normal IHC |
| 24 | 11R-00031 | Familial | 20 | LGMW + distal | Triceps weakness; fingers extensors | 276 | Nonspecific myopathic changes, minimal reduction in dysferlin on IHC |
| 25 | 11R-00230 | Familial | 3 | LGMW | Dilated cardiomyopathy | 8300 | Muscular dystrophy, partial merosin deficiency |
| 26 | 11R-00232 | Familial | 9 | LGMW | Calf muscles hypertrophy | 1380 | Muscular dystrophy, normal IHC |
| 27 | 11R-00308 | Familial | 16 | (Remove Distal) MM | - | 10500 | Muscular dystrophy, absent  dysferlin on IHC |
| 28 | 11R-00337 | Familial | 5 | LGMW | Steroid responsive; wheel chaired at 17 yrs | 2500 | Muscular dystrophy, mild reduction of dystrophin, dysferlin and sarcoglycans on IHC |
| 29 | 11R-00463 | Familial | 3 | LGMW | - | 7722 | Muscular dystrophy, absent α and marked reduction in γ sarcoglycans on IHC |
| 30 | 11R-00643 | Sporadic | 1.5 | LGMW | - | 11173 | Muscular dystrophy, absent all sarcoglycans on IHC |
| 31 | 11R-00745 | Sporadic | 16 | MM | - | 7387 | Muscular dystrophy, absent dysferlin on IHC |
| 33 | 11R-01506 | Familial | 17 | LGMW, MM (Mixed) | - | 17000 | Muscular dystrophy with mild inflammation |
| 34 | 11R-01601 | Familial | 2 | LGMW | No contracture | 7500 | Muscular dystrophy, absent all sarcoglycans on IHC |
| 36 | 11R-02080 | Familial | 9 | LGMW and Facial weakness | Facial and neck weakness | 38 | End-stage muscles |
| 38 | 11R-02618 | Sporadic | 11 | LGMW | - | 2480 | Necrotizing myopathy, normal IHC |
| 39 | 11R-02841 | Familial | 1 | LGMW | Prominent calf muscles | 7920 | Muscular dystrophy with type 2 atrophy, mild reduction of γ-sarcoglycan on IHC |
| 40 | 11R-03100 | Familial | 6 | LGMW | - | 4340 | Muscular dystrophy with mild inflammation, normal IHC |
| 41 | 12R-00001 | Sporadic | 35 | Asymmetrical calves muscles atrophy and weakness | - | 9500 | Muscular dystrophy, normal IHC |
| 42 | 12R-00316 | Familial | 8 | LGMW | Scoliosis; wheel chaired at 30 yrs | 6000 | Necrotizing myopathy, mild reduction in dystrophin and α-sarcoglycanI on IHC |
| 43 | 12R-00468 | Familial | 9 | Ophthalmoplegia, proximal U L weakness | Cardiomyopathy; heart transplant | 300 | Normal muscle with marked mitochondrial proliferation |
| 46 | 12R-01186 | Familial | 8 | LGMW | Cardiomyopathy; wheel chaired at 15 yrs | 3000 | Muscular dystrophy with marked mitochondrial proliferation and COX-negative fibers, absent β-sarcoglycan and dystrophin and marked reduction in other sarcoglycans |
| 48 | 12R-01188 | Sporadic | 20 | LGMW | - | 102 | Necrotizing myopathy, partial loss of  β and  γ -sarcoglycans on IHC |
| 49 | 12R-01189 | Sporadic | 16 | LGMW + MM | - | 8500 | Muscular dystrophy with mild inflammation, absent dysferlin |
| 50 | 12R-01190 | Sporadic | 14 | MM | - | 7800 | Muscular dystrophy with marked mitochondrial proliferation, absent dysferlin on IHC |
| 51 | 12R-02092 | Sporadic | 7 | LGMW | Facial dysmorphic features | 1680 | Myopathic with mitochondrial proliferation |
| 52 | 12R-03343 | Familial | 17 | Distal weakness | - | 7600 | Muscular dystrophy |
| 53 | 13R-00574 | Familial | 10 | LGMW | Achilles tendon contracture | 1520 | Muscular dystrophy |
| 54 | 13R-01080 | Familial | 16 | LGMW | - | 4400 | Muscular dystrophy |
| 55 | 13R-01177 | Familial | 13 | LGMW + MM | Calf hypertrophy in MRI | 8000 | Muscular dystrophy with mild inflammation, absent dysferlin |
| 56 | 14R-00387 | Familial | 15 | LGMW | Mild contraction | 2500 | Myopathic and occasional regenerative fibers. |
| 58 | 14R-0183 | Familial | 21 | LGMW | Dysphagia, bulbar weakness | 285 | Myopathic inclusion bodies |
| 59 | 14R-02300 | Familial | 24 | LGMW | - | 6800 | Muscular dystrophy with mild inflammation |
| 74 | 15R-03389 | Familial | 8 | No weakness (weaking) | Muscle changes | 6900 | Myopathic with mild mitochondrial proliferation |
| 75 | 16NGS-0099 | Sporadic | 8 | LGMW | Contracture at Achilles tendons | 8000 | Muscular dystrophy |
